# Preoperative albumin as a predictor of one-year mortality in patients with fractured neck of femur

**DOI:** 10.1308/003588413X13511609954815

**Published:** 2013-01

**Authors:** WKM Kieffer, CS Rennie, AJ Gandhe

**Affiliations:** Portsmouth Hospitals NHS Trust,UK

**Keywords:** Orthopaedic surgery, Trauma, Femoral neck fractures, Arthroplasty, Fracture fixation

## Abstract

A simple measure to determine one-year mortality following hip fractures has its benefits. Where there is controversy over implant selection, such a scoring system can facilitate the decision-making process. We undertook a retrospective analysis of one-year postoperative mortality of our hip fracture patients and established their admission serum albumin levels to see if there was any correlation between this and one-year mortality. Our results showed one-year mortality was significantly higher (*p*=0.0049) for those patients with a serum albumin of <35g/dl. Of the patients with low albumin, we found that there was no statistical significance between one-year mortality and source of admission (*p*=0.0789). Prefracture serum albumin can be used as a simple predictor of one-year mortality in patients presenting with a fractured neck of femur, thereby aiding operative planning and implant selection with a view to likely survival and possible need for revision.

Fractured neck of femur is a large and growing financial burden for the health budget. Predicted mortality after fractured neck of femur fracture nationally is measured at 20–35%.^1–3^ The mortality for fractured neck of femur can be measured using previously published scoring systems (eg Nottingham Hip Fracture Score [NHFS])^4^ but these can be cumbersome and sometimes impractical when deciding on patient management. A simpler solution would be to use biochemical markers measured routinely on admission.

Intracapsular fractures can be treated with a variety of methods and implants, ranging from fixation to partial and total arthroplasties. While trauma surgeons will have developed their own informal criteria for deciding who is managed with each option, a biochemical predictor of mortality that is quick and easy to interpret would be useful in aiding that decision so that those patients who are likely to live longer do not suffer from the complications associated with the aforementioned implants or have an implant that is easier to revise or modify should the need arise.

In the UK, the National Institute for Health and Clinical Excellence recommends that hemiarthroplasties have an Orthopaedic Data Evaluation Panel rating of 10A,^5^ which is a considerable expense and therefore a financial burden on the health service, making implant selection a vital part of cost control.

Predictors of increased one-year mortality already known from the literature and components of the NHFS include increased age, male sex, low mini-mental state examination score, low haemoglobin levels, presence of malignancy, prefracture institutional residence and increased number of co-morbidities.^4^ While the majority of these factors are calculable on admission, there are some factors that cannot be recorded adequately in the acute setting, thereby limiting their usefulness in operative planning.

Low preoperative serum albumin has been used to predict significantly higher in-hospital mortality and specific postoperative complications^6^ while a state of protein energy malnutrition (low total lymphocyte count and serum albumin; PEM) has been used as a marker of increased 3 and 12-month mortality.^7^ There are few data for preoperative serum albumin alone as a predictor of long-term mortality after fractured neck of femur. The aim of this study was to see if preoperative serum albumin is an accurate predictor of one-year mortality in all patients with fractured neck of femur.

## Methods

All patients admitted to our orthopaedic department with a fractured neck of femur during 2008 were included in this study. The data were collected by our orthogeriatrics team, who collate all the data required for the UK National Hip Fracture Database with follow-up by telephone questionnaire. Normal serum albumin was taken as ≥35g/dl.

Patients’ prefracture mobility was classified as: no aids, one aid, two aids or frame, wheelchair or bedbound. Source of admission was classified as: acute hospital, own home or sheltered housing, residential home, nursing home or longterm care, or other (Table 1). Exclusion criteria included loss to one-year follow-up, no albumin recorded within 72 hours of admission and no recorded data for prefracture residence or mobility.

**Table 1:** Classification of patients within three categories

Albumin	Prefracture mobility	Source of admission
<35g/dl	No aids	Acute hospital
≥35g/dl	One aid	Own home/sheltered housing
	Two aids/frame	Residential/nursing home/long-term care
	Wheelchair/bedbound	Other

**Table 2:** Percentage mortality by admission serum albumin

Albumin	Dead		Total	Mortality
	Yes	No		
<35	84.0	171.0	255.0	32.9%
≥35	74.0	256.0	330.0	22.2%
**Total**	**158.0**	**427.0**	**585.0**	

**Figure 1:**
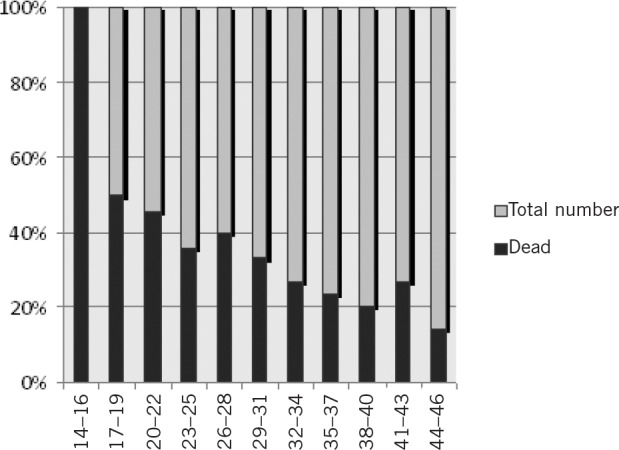
Percentage mortality by serum albumin

This database was interrogated retrospectively to collate the required data. Serum albumin levels for the corresponding admission were obtained by a manual search through the archived database of blood test results at Queen Alexandra Hospital. Statistical analysis was performed by the department’s statistician from the University of Portsmouth using Fisher’s exact test and the Fisher–Freeman–Halton exact test.

## Results

The total number of patients admitted to our department during the year totalled 645, of which 585 met the inclusion criteria. The median age was 84 years and 77.9% of patients were female. Almost half the patients (43.6%, *n*=255) had a serum albumin level of <35g/dl. The median albumin level was 35g/dl. The mortality for patients with an albumin level of <35g/dl was 32.9% while for those patients with an albumin level of ≥35 it was 22.2% (Table 2). Fisher’s exact test showed that mortality was significantly higher in those with a low albumin level (odds ratio: 1.70, 95% confidence interval: 1.16–2.50, *p*=0.0049) (Fig 1).

**Table 3:** Mortality of hypoalbuminaemic patients by source of admission

Admission source	One-year mortality
Acute hospital	4 (50.0%)
Own home/sheltered housing	52 (28.6%)
Residential/nursing home / long-term care	27 (42.9%)
Other	1 (100.0%)

**Table 4:** Mortality of hypoalbuminaemic patients admitted from their own home/sheltered housing by prefracture mobility

Prefracture mobility	One-year mortality
No aid	18 (25.0%)
One aid	18 (27.2%)
Two aids/frame	13 (34.2%)
Wheelchair	2 (66.6%)
Unknown	1 (33.3%)

**Table 5:** Mortality of hypoalbuminaemic patients admitted from residential home/nursing home/long-term care by prefracture mobility

Prefracture mobility	One-year mortality
No aid	17 (29.4%)
One aid	10 (20.0%)
Two aids/frame	26 (57.7%)
Wheelchair	7 (42.9%)
Unknown	3 (66.6%)

For those patients with low albumin levels, the source of admission is detailed in Table 3. There was no statistically significant difference in those patients with a low albumin level admitted from different sources (Fisher–Freeman– Halton exact test, *p*=0.0789).

Among those patients admitted from their own or a sheltered home (Table 4), there was no significant difference in prefracture mobility and one-year mortality (Fisher–Freeman–Halton exact test, *p*=0.4432). Neither was there a statistically significant difference in one-year mortality among those patients admitted from a residential home, nursing home or long-term care (Fisher–Freeman– Halton exact test, *p*=0.1616) (Table 5).

Data comparing patients with low albumin levels according to source of admission and prefracture mobility for those patients admitted from ‘acute hospitals’ and ‘other’ unfortunately provided too small numbers to provide meaningful statistical data.

## Discussion

It is known that patients presenting with a fractured neck of femur are at risk of PEM.^8^ The extent to which this occurs is documented as well as short-term evidence of increased mortality in patients with low serum albumin.^6^ Elderly patients undergoing surgery for a fractured neck of femur are also known to suffer from protein depletion and metabolic stress following fracture and fixation,^9^ and are less likely to recover their independence should they be in a state of PEM.^10^ O’Daly *et al* have already described the link between mortality and PEM.^7^ However, rather than the need to calculate a patient’s degree of PEM, we have found that the single measure of albumin is a very accurate predictor of one-year mortality as a surrogate marker of PEM, avoiding the need to calculate or measure other biochemical values.

In a busy trauma unit such as our own, where we see over 600 fractured neck of femur patients each year, difficult calculations can be cumbersome to perform quickly for each individual. With an increasing drive for surgery to be carried out within 36 hours of admission, information such as prefracture mobility is not always available to guide implant selection, especially if the patient has cognitive impairment.

Our rationale for the need to establish the accuracy of a single biochemical test as a predictor of one-year mortality stems from a desire to tailor our practice towards patients’ needs. As the population ages, functional demands will inevitably increase and patients may outlive their prostheses. If this happens, then more expensive, modular implant systems may be beneficial over the traditional monoblock hemiarthroplasties often used, especially when revisions are to be contemplated.

In addition, there is increasing evidence that these patients are better treated with total hip arthroplasty.^11,12^ An ability to predict those patients who have high one-year mortality would assist in deciding which implant to select as those with poorer prognoses are unlikely to reap the advantages of such expensive implants. Ideally, all patients should receive the best implant available at the time of surgery. Financial constraints, however, can prevent this from being viable in a busy trauma unit so an ability to match the patients who will best take advantage of more expensive modular hemiarthroplasties is useful.

While this is a useful marker, there is no suggestion of using it as the sole discriminator for implant selection as other factors have to be taken into account such as age, prefracture mobility, functional demands and local cost implications. Although these variables are important in determining quality of life, we have also shown that they are non-significant with regard to mortality.

## Conclusions

A low serum albumin level on admission is a useful sole indicator of increased one-year mortality for patients presenting with a fractured neck of femur (*p*=0.0049). Source of admission and prefracture mobility, however, appear to be non-significant markers of mortality although they still remain important for implant selection and operative planning. Serum albumin should therefore be measured routinely on admission for all fractured neck of femur patients to aid decision making with regard to operative plan.
